# Taxonomic novelties of *Dothiorella* and additions to Botryosphaeriaceae from Yunnan, China

**DOI:** 10.3897/mycokeys.130.183564

**Published:** 2026-03-30

**Authors:** Achala R. Rathnayaka, Jianwei Liu, Zhuyue Yan, Shimei Yang, Jing Yuan, Xiaofei Shi, Shanping Wan, Dongqin Dai, Indunil C. Senanayake, Fuqiang Yu

**Affiliations:** 1 Yunnan Key Laboratory for Fungal Diversity and Green Development, Germplasm Bank of Wild Species, Kunming Institute of Botany, Chinese Academy of Sciences, Kunming 650201, Yunnan Province, China Center for Yunnan Plateau Biological Resources Protection and Utilization, College of Biology and Food Engineering, Qujing Normal University Qujing China; 2 Yunnan International Joint Laboratory of Fungal Sustainable Utilization in South and Southeast Asia, Germplasm Bank of Wild Species, Kunming Institute of Botany, Chinese Academy of Sciences, Kunming 650201, Yunnan Province, China Yunnan International Joint Laboratory of Fungal Sustainable Utilization in South and Southeast Asia, College of Biology and Food Engineering, Qujing Normal University Qujing China; 3 Key Laboratory of Chemistry in Ethnic Medicinal Resources, School of Ethnic Medicine, Yunnan Minzu University, Kunming 650500, Yunnan Province, China Yunnan International Joint Laboratory of Fungal Sustainable Utilization in South and Southeast Asia, Germplasm Bank of Wild Species, Kunming Institute of Botany, Chinese Academy of Sciences Kunming China; 4 College of Resources and Environment, Yunnan Agricultural University, Kunming 650204, Yunnan Province, China Yunnan Key Laboratory for Fungal Diversity and Green Development, Germplasm Bank of Wild Species, Kunming Institute of Botany, Chinese Academy of Sciences Kunming China; 5 Center for Yunnan Plateau Biological Resources Protection and Utilization, College of Biology and Food Engineering, Qujing Normal University, Qujing 655099, China College of Resources and Environment, Yunnan Agricultural University Kunming China; 6 Yunnan International Joint Laboratory of Fungal Sustainable Utilization in South and Southeast Asia, College of Biology and Food Engineering, Qujing Normal University, Qujing 655099, China Key Laboratory of Chemistry in Ethnic Medicinal Resources, School of Ethnic Medicine, Yunnan Minzu University Kunming China

**Keywords:** 2-new species, 2-new host records, Dothideomycetes, morphology, phylogeny

## Abstract

Botryosphaeriaceae is the largest family in Botryosphaeriales, consisting of 22 genera. Members of this family have cosmopolitan distribution, occurring in various life modes: endophytic, saprobic and pathogenic, mainly associated with plants. This study aims to investigate plant-associated saprobic microfungi in Yunnan, China. Here, we establish two novel species in *Dothiorella*, namely *D. fusiformis* and *D. swieteniae*. Both sexual and asexual morphs are provided for *D. fusiformis*. Additionally, we report two new host records of *Barriopsis archontophoenicis* S. Konta, Boonmee & K.D. Hyde and *D. camelliae* W.Li Li & Jian K. Liu. Taxonomic boundaries were confirmed based on morphological characteristics coupled with phylogenetic analyses using maximum likelihood (ML) and Bayesian inference (BI) analyses of combined SSU, LSU, ITS, *β-tubulin* and *tef*1-α sequences. Furthermore, the detailed illustrations, comprehensive descriptions, and taxonomic justifications are provided for each species. This study expands our understanding of the taxonomy, diversity and ecological relationships of Botryosphaeriaceae species in China by introducing new species and reporting new host and geographic records.

## Introduction

Botryosphaeriaceae was established by Theissen and Sydow (1918), with *Botryosphaeria* Ces. & De Not as the type genus. Over the years, Botryosphaeriaceae and its genera have undergone several taxonomic revisions. Phillips et al. (2008) examined the species with brown ascospores and introduced five more genera to Botryosphaeriaceae, providing accurate classification of *Diplodia* and *Lasiodiplodia*. Damm et al. (2007) showed that *Aplosporella* belongs to Botryosphaeriaceae, while Rojas et al. (2008) confirmed that *Endomelanconiopsis* is also part of the same family. Furthermore, Phillips and Alves (2009) considered *Melanops* as an additional genus in Botryosphaeriaceae. Liu et al. (2012) included 29 genera in this family based on morpho-molecular data. Comprehensive descriptions and keys for 17 genera in the family were provided by Phillips et al. (2013). Later, 24 well-defined genera were accepted in Botryosphaeriaceae based on morphology and molecular evidence (Burgess et al. 2019; Garcia et al. 2021). Currently, 22 genera are included in this family, forming clearly separate monophyletic clades. Botryosphaeriaceae represents the largest family within Botryosphaeriales (Zhang et al. 2021; Wijayawardene et al. 2022).

Species of Botryosphaeriaceae have a worldwide distribution, inhabiting a wide range of monocotyledonous and dicotyledonous hosts. They occur on different substrates viz. woody branches, leaves, stems, grass culms, twigs, and in the thalli of lichens (Barr 1987; Denman et al. 2000; Mohali et al. 2007; Lazzizera et al. 2008; Marincowitz et al. 2008). These species have been reported as endophytes (Slippers and Wingfield 2007), pathogens, opportunistic pathogens (Chethana et al. 2016), or saprobes (Schoch et al. 2006), in agricultural crops, ornamental plants, and forest hosts, primarily in terrestrial ecosystems (Batista et al. 2021). Many Botryosphaeriaceae members, specifically species of *Botryosphaeria*, *Diplodia*, *Dothiorella*, *Lasiodiplodia*, and *Neofusicoccum*, have been recognized as pathogens that cause blights, cankers, dieback diseases as well as fruit-rot diseases in ecologically and economically important plants in forests and agricultural fields worldwide (Phillips et al. 2013; Slippers et al. 2013; Zhou et al. 2015; Phillips et al. 2019).

Saccardo (1880) introduced *Dothiorella* with *D. pyrenophora* Berk. ex Sacc. as the type species. Both sexual and asexual morphs have been described in this genus. The sexual morph is characterized by erumpent or superficial ascomata, bitunicate, fissitunicate asci and pigmented, and septate ascospores, while asexual morph exhibits immersed, erumpent conidiomata with hyaline, holoblastic conidiogenous cells (Phillips et al. 2013). Interestingly, the conidia become brown and 1-septate while still attached to the conidiogenous cells (Phillips et al. 2005; Dissanayake et al. 2016; Hongsanan et al. 2020). *Dothiorella* species occur on a wide range of hosts as endophytes, saprobes or pathogens (Phillips et al. 2013; Dissanayake et al. 2016; Jayawardena et al. 2019). The majority of *Dothiorella* species are plant pathogens or saprobes on a variety of woody hosts and they have a cosmopolitan distribution (Li et al. 2014). Currently, 348 species are listed in Index Fungorum (2026), while 336 epithets are listed under *Dothiorella* in Species Fungorum (2026). However, molecular data are available for only 60 species in GenBank (Zhang et al. 2025).

*Barriopsis* was introduced with *B. fusca* (N.E. Stevens) A.J.L. Phillips, A. Alves & Crous as the type species (Phillips et al. 2008). The sexual morph of this genus is characterized by bitunicate asci and brown ascospores that are widest in the middle, and lack terminal apiculi (Phillips et al. 2008). Lasiodiplodia-like asexual morph of this genus is characterized by initially hyaline, aseptate, thick-walled conidia, becoming dark brown and septate with irregular longitudinal striations at maturity (Phillips et al. 2008). However, compared to *Lasiodiplodia*, conidia of *Barriopsis* are striate at the early stage and striations visible on hyaline conidia while attached to conidiogenous cells (Abdollahzadeh et al. 2009). Currently, seven *Barriopsis* species are listed in Index Fungorum (2026).

Botryosphaeriaceae species occur worldwide and have different lifestyles, making further taxonomic and ecological analyses prerequisites. During our investigations of plant-associated microfungi in Yunnan, China, we collected and isolated five Botryosphaeriaceae taxa. In this study, we introduce two novel *Dothiorella* species with two new host records of *Barriopsis* and *Dothiorella*. Morphological illustrations, detailed descriptions, multigene phylogenetic analyses with maximum likelihood (ML) and Bayesian inference (BI) analyses confirm the phylogenetic placement of these newly isolated fungal taxa. Taxonomic notes with appropriate justifications for each taxon were also provided.

## Materials and methods

### Specimen collections, morphological studies and isolations

Fresh fungal samples were collected in different forest sites in Yunnan, China in July – September 2025. Specimens were enclosed in zip-loc plastic bags and brought to the laboratory. The samples were examined for morphology following the methodology described in Rathnayaka et al. (2024). Both macro- and micro- morphological characters were examined using a LEICA IVESTA 3 stereomicroscope (Leica Microsystems, Germany), and LEICA DM2500 compound microscope (Leica Microsystems, Germany), respectively and characters were photographed with a Canon 550D digital camera fitted to the compound microscope. Measurements were made with ZEN2 (blue edition) software and calculated with the Tarosoft (R) Image Frame Work program. Photographs were processed, and photo plates were prepared with Adobe Photoshop CS3 Extended version 10.0 software (Adobe Systems, USA).

Single spore isolations were carried out for fungal isolation as described in Senanayake et al. (2020) by using potato dextrose agar (PDA) media plates and incubated at 25 °C. Pure cultures were obtained by sub culturing, and culture characters were recorded after one week. The dried specimens were deposited at the fungarium of the Kunming Institute of Botany Academia Sinica (**HKAS**) and living cultures were deposited at the Germplasm Bank of Wild Species Culture Collection (**GBWSCC**). Index Fungorum numbers were obtained (http://www.indexfungorum.org) for the novel taxa.

### DNA extraction, PCR amplification and sequencing

Genomic DNA was extracted from fresh mycelium (50–100 mg) using TIANGEN Plant Genomic DNA Kit (Tiangen Biotech, Beijing) by following the manufacturer’s instructions. Polymerase chain reactions (PCR) were performed for a small subunit of nuclear ribosomal RNA (SSU), a large subunit of nuclear ribosomal RNA (LSU), internal transcribed spacers region (ITS), partial translation elongation factor 1-α gene (*tef*1-α) and beta-tubulin (*β-tubulin*) genes. The relevant PCR thermal cycles with the conditions are given in Table 1. PCR reactions were carried out in a final volume of 50 μl, which contained 21 µl of deionized water, 25 µl of 2× Power Taq PCR Master Mix (CWBIO, China), 1 μl of each forward and reverse primers and 2 μl of genomic DNA. PCR products were visualized on 1.5% agarose gels containing the Safeview DNA stain (Biosharp Life Sciences, China) and sequenced at Sangon Biotech sequencing laboratory (China). The remaining extracted DNA was stored at 4 °C for a short-term and –20 °C for long- term storage. Newly generated nucleotide sequences were deposited in GenBank (Table 2).

**Table 1.** Gene regions used in the study with PCR primers, conditions and references.

Gene region	Primers	PCR conditions	Reference(s)
SSU	NS1/NS4	95 °C, 3 min, (95 °C, 30 s, 55 °C, 50 s, 72 °C, 30 s) × 35 cycles, 72 °C, 10 min	White et al. (1990)
LSU	LR0R/LR5	95 °C: 3 min, (95 °C: 30 s, 55 °C:50 s, 72 °C: 30 s) × 35 cycles, 72 °C, 10 min	Vilgalys and Hester (1990)
ITS	ITS4/ITS5	95 °C: 3 min, (95 °C: 30 s, 55 °C:50 s, 72 °C: 30 s) × 35 cycles, 72 °C, 10 min	White et al. (1990)
*tef*1-α	EF1–728F/ EF1–986R	95 °C: 5 min, (94 °C: 30 s, 55 °C:45 s, 72 °C: 90 s) × 30 cycles, 72 °C, 10 min	Carbone and Kohn (1999)
*β-tubulin*	Bt2a/Bt2b	95 °C: 5 min, (94 °C: 30 s, 55 °C:45 s, 72 °C: 90 s) × 30 cycles, 72 °C, 10 min	Glass and Donaldson (1995)

**Table 2.** Taxa used in the phylogenetic analyses and their GenBank accession numbers. Newly introduced taxa and two extant species generated in this study are indicated in blue and type strains are indicated in bold.

Species	Strain no.	GenBank accession no.				
		SSU	LSU	ITS	*tef*1-α	*β-tubulin*
***Alanphillipsia aloeicola***	**CBS 138896**	N/A	MH878642	KP004444	N/A	–
***Alanphillipsia aloeigena***	**CPC 21286**	N/A	KF777193	KF777137	N/A	–
***Barriopsis archontophoenicis***	**MFLUCC 14-1164**	NG 063609	KX235307	KX235306	N/A	–
*Barriopsis archontophoenicis*	MFLU 20-0523	MT876121	MT876523	MT876524	MT897877	–
*Barriopsis archontophoenicis*	GBWSCCF120264	PX498709	PX498707	PX498655	PX562762	N/A
*Barriopsis archontophoenicis*	HKAS 151750	N/A	PX498708	PX498656	N/A	N/A
***Barriopsis caryotae***	**UESTCC:23.0407**	N/A	PQ166771	PQ181882	PQ166410	PQ390669
***Barriopsis iraniana***	**IRAN 1448C**	KF766231	KF766318	FJ919663	FJ919652	–
*Barriopsis iraniana*	IRAN1450C	–	–	FJ919667	FJ919656	–
***Barriopsis menglaensis***	**KUMCC 21-0578**	PQ206508	PQ206506	PQ206504	PQ218693	–
***Barriopsis stevensiana***	**CBS 174.26**	KF766230	DQ377857	EU673330	EU673296	–
*Barriopsis stevensiana*	MFLUCC 11-0420	MN582689	MN582755	MN582740	–	–
***Barriopsis tectonae***	**MFLUCC 12-0381**	–	–	KJ556515	KJ556516	–
*Barriopsis thailandica*	MFLUCC 14-1190	–	–	KY115675	KY115676	–
***Dothiorella acacicola***	**CPC 26349**	–	KX228320	NR_145255	KX228376	N/A
*Dothiorella acericola*	KUMCC 18-0137	–	N/A	MK359449	MK361182	N/A
***Dothiorella albiziae***	**MFLUCC 22-0057**	–	ON751764	ON751762	ON799588	ON799590
*Dothiorella alpina*	CGMCC 3.18001	–	N/A	KX499645	KX499651	N/A
***Dothiorella baihuashanensis***	**CFCC 58549**	–	N/A	OQ651168	OQ692934	OQ692928
***Dothiorella brevicollis***	**CMW 36463**	–	NG_069098	NR_111703	JQ239390	JQ239371
***Dothiorella camelliae***	**CGMCC 3.24158**	–	N/A	OQ190531	OQ241464	OQ275064
*Dothiorella camelliae*	GBWSCCF110208	–	PX499329	PX499328	N/A	PX562763
*Dothiorella capri-amiss*	CBS:121763	–	KX464301	EU101323	EU101368	KX464850
*Dothiorella casuarini*	CBS 120688	–	MH874647	DQ846773	DQ875331	N/A
***Dothiorella chiangmaiensis***	MFLU 22-0161	–	N/A	NR_187083	OP614929	N/A
*Dothiorella citricola*	ICMP16828	–	EU673242	EU673323	EU673290	EU673145
***Dothiorella citrimurcotticola***	CGMCC 3.20394	–	N/A	MW880661	MW884164	MW884193
***Dothiorella diospyricola***	**CBS 145972**	–	N/A	MT587398	MT592110	MT592581
***Dothiorella dulcispinae***	CMW 36460	–	NG_069097	NR_111702	JQ239387	JQ239373
***Dothiorella eriobotryae***	**CBS 140852**	–	MH878199	KT240287	KT240262	MT592582
***Dothiorella franceschinii***	**CBS 147722**	–	N/A	NR_198362	OQ067247	N/A
***Dothiorella heterophyllae***	**CMW46458**	–	N/A	MN103794	MH548348	MH548324
***Dothiorella fusiformis***	**GBWSCCF120274**	–	PX727435	PX727439	N/A	PX730059
*Dothiorella fusiformis*	HKAS 151748	–	PX727436	PX727440	N/A	PX730060
*Dothiorella fusiformis*	**GBWSCCF120275**	–	PX727437	PX727441	N/A	PX730061
*Dothiorella fusiformis*	HKAS 151752	–	PX727438	PX727442	N/A	PX730062
***Dothiorella hortiarborum***	**CFCC 70756**	–	N/A	PP188524	PP723042	PP566662
***Dothiorella iranica***	**IRAN1587C**	–	NG_228749	KC898231	KC898214	KX464856
*Dothiorella koae*	CMW 48017	–	N/A	MH447652	MH548338	MH548327
***Dothiorella lampangensis***	MFLUCC 18-0232	–	N/A	NR_163336	MK340869	MK412874
*Dothiorella longicollis*	CBS 122068	–	NG_069903	EU144054	EU144069	KF766130
*Dothiorella macadamiae*	SDBR-CMU513	–	N/A	PQ699725	PQ758593	PQ736694
***Dothiorella magnoliae***	**CFCC 51563**	–	N/A	NR_191055	KY213686	N/A
***Dothiorella mangifericola***	**CBS 121760**	–	N/A	EU101290	EU101335	KX464877
*Dothiorella mangifericola*	IRAN1584C	–	N/A	KC898221	KC898204	N/A
***Dothiorella moneti***	**MUCC505**	–	EF591937	EF591920	EF591971	EF591954
***Dothiorella. oblonga***	**CBS 121765**	–	KX464318	NR_137689	EU101345	N/A
***Dothiorella obovata***	**MFLUCC 22-0058**	–	ON751765	ON751763	ON799589	ON799591
***Dothiorella ovata***	**MFLUCC 23-0035**	–	OR020691	OR052059	OR030456	OR030474
*Dothiorella plurivora*	IRAN1557C	–	N/A	KC898225	KC898208	N/A
***Dothiorella pretoriensis***	**CMW 36480**	–	JQ239418	JQ239405	JQ239392	JQ239376
*Dothiorella prunicola*	CAP187	–	EU673232	EU673313	EU673280	EU673100
*Dothiorella pugeense*	SICAUCC 23-0142	–	PP862534	PP844870	PV220917	PV220918
***Dothiorella rhamni***	**MFLUCC 14-0902**	–	KU246382	KU246381	MF398945	N/A
***Dothiorella rosacearum***	**MFLUCC 23-0014**	–	OR052043	OR052061	OR030458	OR030476
***Dothiorella rosulata***	**CBS 121760**	–	NG_069178	NR_136991	EU101335	KX464877
***Dothiorella sarmentorum***	**CBS 128309**	–	MH876298	HQ288218	MT592106	MT592577
***Dothiorella santali***	**MUCC509**	–	EF591941	EF591924	EF591975	EF591958
***Dothiorella saprophytica***	MFLUCC 23-0210	–	N/A	OR527239	OR532455	OR532454
*Dothiorella sarmentorum*	MFLUCC 17-0242	–	KY815014	KY797637	N/A	MT592585
*Dothiorella sarmentorum*	CBS 115041	–	AY928053	AY573202	AY573222	EU673096
*Dothiorella sarmentorum*	MFLUCC 17-0951	–	N/A	MG828897	MG829267	MT592592
***Dothiorella sarmentorum***	**CBS 392.80**	–	N/A	KX464133	KX464626	KX464897
*Dothiorella sarmentorum*	IRAN1579C	–	N/A	KC898234	KC898217	N/A
*Dothiorella sarmentorum*	IRAN1583C	–	OQ164838	KC898236	KC898219	N/A
*Dothiorella sarmentorum*	MFLUCC 13-0498	–	N/A	KJ742379	KJ742382	N/A
*Dothiorella sarmentorum*	CBS 725.79	–	N/A	KX464130	KX464622	KX464888
*Dothiorella sarmentorum*	**IMI 63581b**	–	NG_042411	AY573212	AY573235	MT592612
***Dothiorella septata***	MFLUCC 23-0039	–	OR020695	OR020942	OR030462	OR030480
*Dothiorella striata*	ICMP 16819	–	N/A	EU673320	EU673287	EU673142
***Dothiorella striata***	**DAR80992**	–	N/A	KJ573643	KJ573640	N/A
***Dothiorella swieteniae***	**GBWSCCF120263**		PX499330	PX494348	N/A	N/A
*Dothiorella swieteniae*	HKAS 151749		PX499331	PX494349	N/A	N/A
*Dothiorella tectonae*	MFLUCC12-0382	–	N/A	KM396899	KM409637	KM510357
***Dothiorella thailandica***	**MFLUCC 11-0438**	–	NG_042725	NR_111794	JX646861	JX646844
*Dothiorella thripsita*	BRIP 51876	–	KX464338	KJ573642	KJ573639	KJ577550
***Dothiorella ulmacea***	**CBS 138855**	–	KR611899	KR611881	KR611910	KR611909
***Dothiorella uruguayensis***	**CBS 124908**	–	MH874932	NR_156208	N/A	KX464886
***Dothiorella vinea-gemmae***	**B116-3**	–	N/A	KJ573644	KJ573641	KJ577552
***Dothiorella viticola***	**WA10NO01**	–	N/A	HM009376	HM800511	HM800519
*Dothiorella viticola*	WA10NO02	–	N/A	HM009377	HM800512	HM800520
*Dothiorella yunnana*	CGMCC 3.18000	–	N/A	KX499644	KX499650	N/A
*Dothiorella zanthoxyli*	CGMCC 3.24159	–	N/A	OQ190536	OQ241468	OQ275069
***Lasiodiplodia crassispora***	**CBS 118741**	EU673190	DQ377901	DQ103550	EU673303	N/A
***Lasiodiplodia avicenniae***	**CMW41467**	N/A	N/A	KP860835	KP860680	N/A
***Neofusicoccum luteum***	**CBS 562.92**	–	KX464430	KX464170	KX464690	KX464968
***Neofusicoccum luteum***	**CMW 41365**	–	N/A	NR_147360	KP860702	KP860779

N/A: Sequences not available; “–”: Sequences not used for analyses.

### Phylogenetic analyses

The quality of the sequence chromatograms was checked manually using the BioEdit v 7.0.9.0 software (Hall 1999). Newly generated sequences were initially subjected to the BLASTn search engine at NCBI to observe more similar searches and related literature was referred to the recent taxonomic updates (Rathnayaka et al. 2022; Wang et al. 2025; Zhou et al. 2025). Sequences generated from this study and isolates retrieved from the GenBank are shown in Table 2. Each locus (SSU, LSU, ITS, *tef*1-α and *β-tubulin*) was aligned individually with MAFFT 6.864b (Katoh et al. 2019) and trimmed using trimAl v1.2 software in gappyout mode (Capella-Gutiérrez et al. 2009). Single gene and multi-gene aligned datasets were analysed separately using ML and BI. MrModeltest v. 2.2 (Nylander 2004) under the AIC (Akaike Information Criterion) implemented in PAUP v. 4.0b10 to find out the best fit models for BI and ML analyses. The GTR+G model was selected as the best model for both ML and BI analyses for each gene region.

The ML analyses were performed using IQ-Tree with bootstrap support obtained from 1,000 pseudoreplicates (Nguyen et al. 2015; Chernomor et al. 2016). The BI analyses were conducted with MrBayes v. 3.2.6 (Ronquist et al. 2012). The Markov Chain Monte Carlo (MCMC) algorithm of six chains was initiated for 1,000,000 generations. The trees were sampled at every 1000^th^ generation, resulting in 1,000 trees. The first 10% of trees were discarded as the burn-in phase, while the remaining 90% were used to calculate the posterior probabilities (PP) in the majority rule consensus tree. Phylograms were visualized with the FigTree v1.4.0 program (Rambaut 2012) and reorganized in Microsoft PowerPoint (2010).

## Results

### Phylogenetic analyses

**Figure 1.**
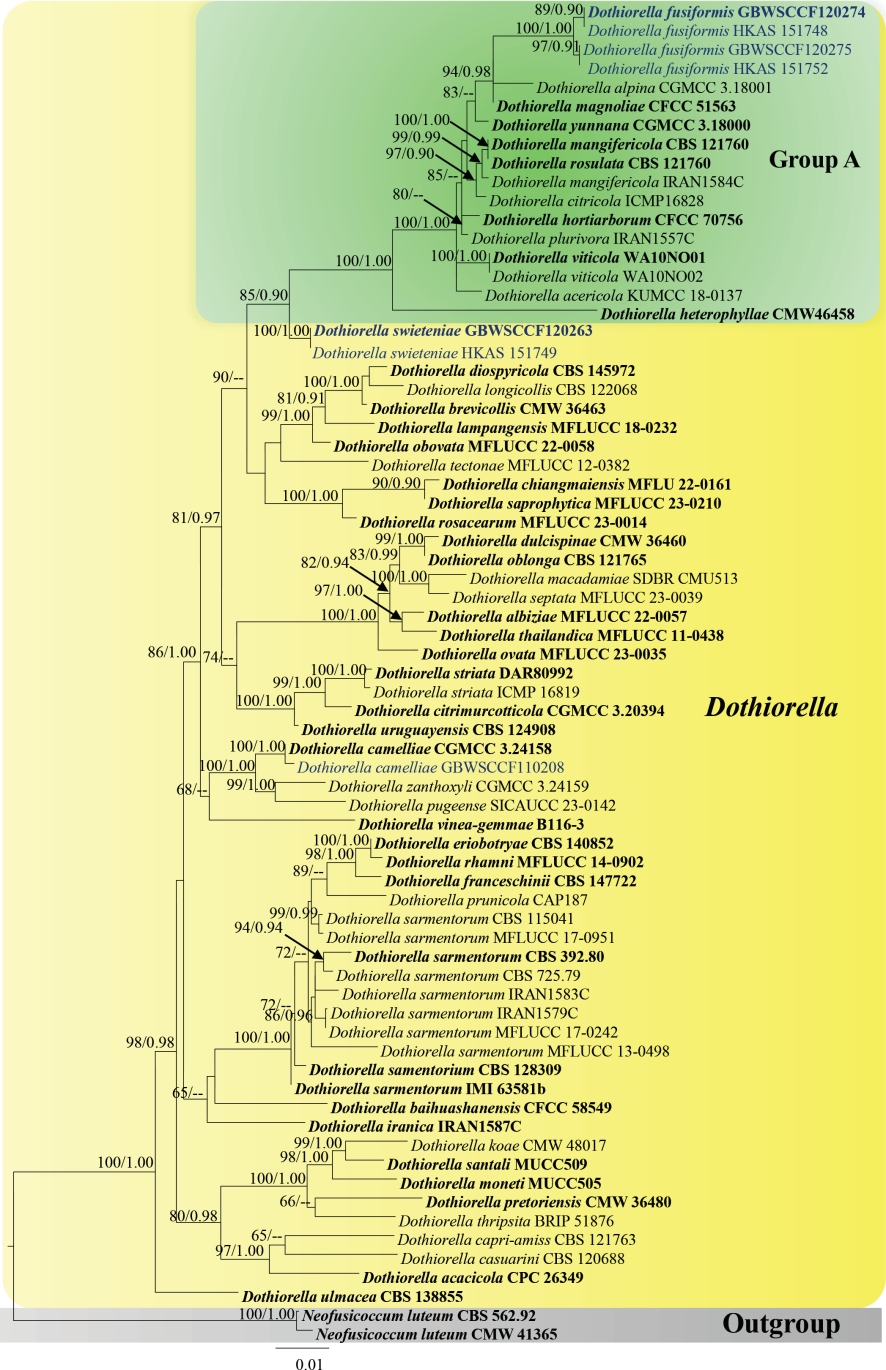
Phylogenetic tree generated from ML analysis based on combined dataset of LSU, ITS, *tef*1-α and *β-tubulin*. The tree is rooted to *Neofusicoccum luteum* (CMW 4165 and CBS 562.26). Bootstrap support values for ML ≥ 65% and Posterior probabilities (PP) ≥ 0.90 are noted at the nodes. Strain numbers are noted after the species names. Strains isolated in this study are represented as blue and type strains are in bold.

For *Dothiorella*, the combined LSU, ITS, *tef*1-α and *β-tubulin* dataset including 71 taxa was used with *Neofusicoccum luteum* (CBS 562.92 and CMW 41365) as the outgroup taxa. Related sequences were obtained from GenBank and related literature (Rathnayaka et al. 2022; Wang et al. 2025; Zhou et al. 2025). Final sequence alignment consisted of 2,015 characters including gaps (LSU = 861, ITS = 567, *tef*1-α = 157, *β-tubulin* = 430). Both ML and BI analyses exhibit similar tree topologies. The best scoring RAxML tree with a final likelihood value of -8660.703 is presented (Fig. 1). The matrix of combined dataset included 579 distinct alignment patterns, with 25.77% undetermined characters or gaps. Estimated base frequencies were obtained as follows: A = 0.218, C = 0.279, G = 0.274, T = 0.229; substitution rates AC = 1.0, AG = 1.97806, AT = 1.0, CG = 1.0, CT = 4.75260, GT = 1.0; gamma distribution shape parameter α = 0.604. In BI analysis, 10,001 trees resulted after 1,000,000 generations. The first 1000 trees, representing the burn-in phase, were discarded, while the remaining 9001 trees were used to calculate posterior probabilities in the majority rule consensus tree. The average standard deviation of split frequencies was 0.01 after 1,000,000 generations of runs. According to the phylogenetic analyses, our new strains (GBWSCCF120274, HKAS 151748, GBWSCCF120275, HKAS 151752, GBWSCCF120263 and HKAS 151749) formed separate clades within *Dothiorella*.

**Figure 2.**
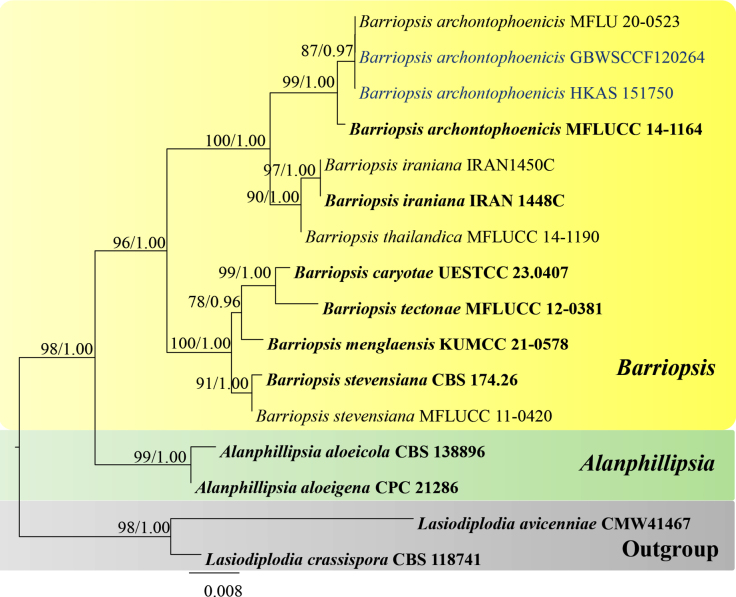
Phylogram generated from ML analysis based on combined dataset of SSU, LSU, ITS and *tef*1-α. The tree is rooted to *Lasiodiplodia crassispora* (CBS 118741) and *L. avicenniae* (CMW 41467). Bootstrap support values for ML ≥ 65% and Bayesian posterior probabilities (PP) ≥ 0.90 are noted at the nodes. Strain numbers are noted after the species names. Strains isolated in this study are represented as blue and type strains are in bold.

For *Barriopsis*, the combined dataset (SSU, LSU, ITS and *tef*1-α) consisted of 16 taxa was used with *Lasiodiplodia crassispora* (CBS 118741) and *L. avicenniae* (CMW 41467) as the outgroup taxa. Related sequences were obtained from GenBank and Dong et al. (2025). After aligning, the dataset comprised 2,708 characters including gaps (SSU = 1,024, LSU = 849, ITS = 500, *tef*1-α = 335). Topology of the BI tree (not shown) was similar to the ML tree. The best scoring RAxML tree, with a final likelihood value of -5520.171 is shown in Fig. 2. The matrix had 295 distinct alignment patterns, with 10.8% undetermined characters or gaps. Estimated base frequencies were as follows: A = 0.25, C = 0.25, G = 0.25, T = 0.25; substitution rates AC = 1.0, AG = 2.89532, AT = 1.0, CG = 1.0, CT = 6.81738, GT = 1.0; gamma distribution shape parameter α = 0.176413. In BI analysis, the average standard deviation of split frequencies was 0.001 after 1,000,000 generations of runs.

### Taxonomy

#### Dothiorella fusiformis

Taxon classificationFungiBotryosphaerialesBotryosphaeriaceae

Rathnayaka & F. Q. Yu sp. nov.

5E17FAFB-35C3-5A95-8F29-CE0A5AEBD945

Index Fungorum: IF904781

Figs 3, 4

##### Etymology.

The epithet refers to fusiform-shaped ascospores.

##### Holotype.

HKAS 151748.

##### Description.

***Saprobic*** on dead twigs of *Thalictrum* sp. ***Sexual morph*: *Ascomata*** 190–250 μm high × 190–225 μm diam. (x̄ = 220 × 200 μm, n = 10), immersed to erumpent through host tissue, globose, solitary and scattered, black, unilocular. ***Peridium*** 20–35 μm wide, composed of two layers, outer layer consists of thick-walled, dark brown to brown cells of *textura angularis*, inner layer composed of thin-walled, pale brown to hyaline cells of *textura angularis*. ***Hamathecium*** comprising numerous 3–5 μm wide, hyaline, septate, unbranched pseudoparaphyses. ***Asci*** 80–91 × 17–20 μm (x̄ = 87 × 19 μm, n = 30), 8 or 4-spored, bitunicate, fissitunicate, cylindrical-clavate to clavate, short-pedicellate, apically rounded, with ocular chamber. ***Ascospores*** 13–18 × 6–10 μm (x̄ = 16 × 5 μm, n = 30), length/width (l/w) ratio = 3.2, overlapping 2–3-seriate, broadly fusiform, straight or slightly curved, initially hyaline and aseptate, becoming dark brown and 1-septate at maturity, slightly constricted at the septum, rough-walled. ***Asexual morph***: *Saprobic* on the dead twigs of *Thalictrum* sp. Coelomycetous. ***Conidiomata*** 295–325 μm high × 310–375 μm diam. (x̄ = 310 × 350 μm, n = 10), pycnidial, solitary, globose, formed in uniloculate stromata, immersed to erumpent through host tissue. ***Conidiomatal wall*** 20–38 μm diam., 4–6 layers, consists of outer layers of thick-walled, dark brown to brown cells of *textura angularis*, inner layers consists of thin-walled, pale brown to hyaline cells of *textura angularis*. ***Conidiophores*** usually reduced to conidiogenous cells. ***Conidiogenous cells*** 10–4 μm high × 3–5 μm diam. (x̄ = 6 × 4 μm, n = 15), lining the pycnidial cavity, holoblastic, hyaline, cylindrical, discrete, determinate, smooth-walled. ***Conidia*** 10–18 × 5–7 μm (x̄ = 15 × 6 μm, n = 25, l/w = 2.5), oblong to ellipsoidal, straight or slightly curved, rounded at both ends, initially hyaline and aseptate, becoming dark brown often while attached to conidiogenous cell, slightly constricted at the septum, guttulate.

##### Culture characteristics.

**Figure 3.**
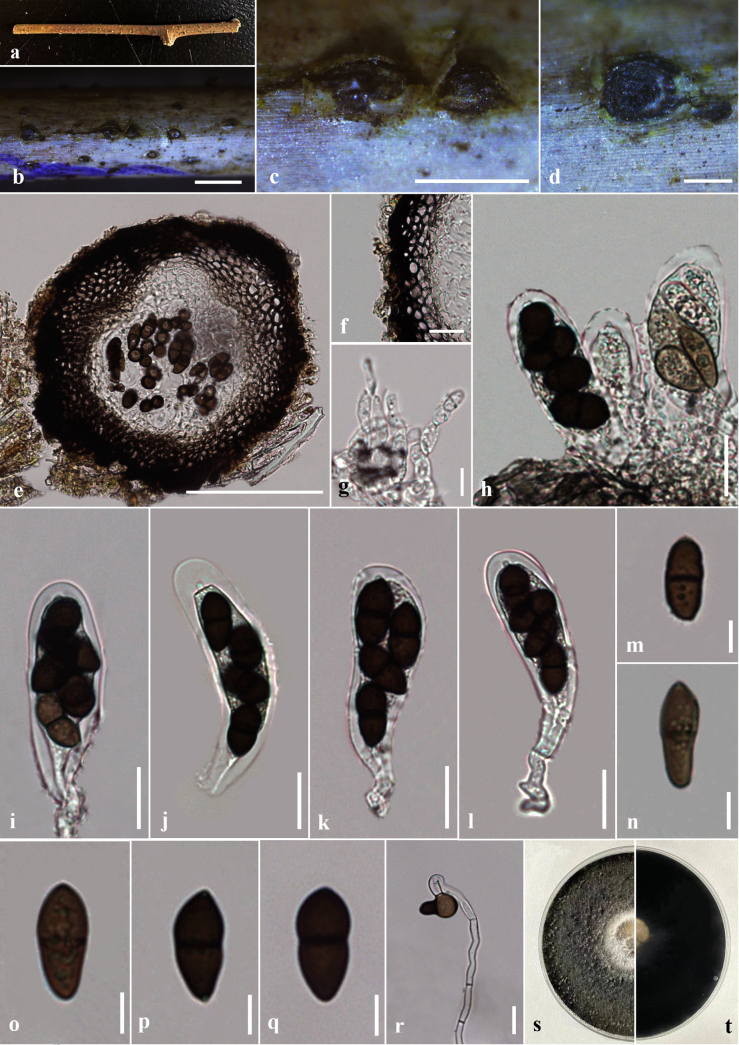
*Dothiorella fusiformis* (HKAS 151748, Holotype, sexual morph) on dead twig of *Thalictrum* sp. **a**. Natural host substrate; **b, c**. Appearance of ascostromata on the host; **d, e**. A section through an ascoma; **f**. Peridium; **g**. Pseudoparaphyses; **h–l**. Asci; **m–q**. Ascospores; **r**. A germinated ascospore; **s, t**. Colony on PDA (**s**. Upper; **t**. Lower views). Scale bars: 1 mm (**b**); 500 μm (**c**); 200 μm (**d**); 100 μm (**e**); 20 μm (**f, h–l**); 10 μm (**g, r**); 5 μm (**m–q**).

**Figure 4.**
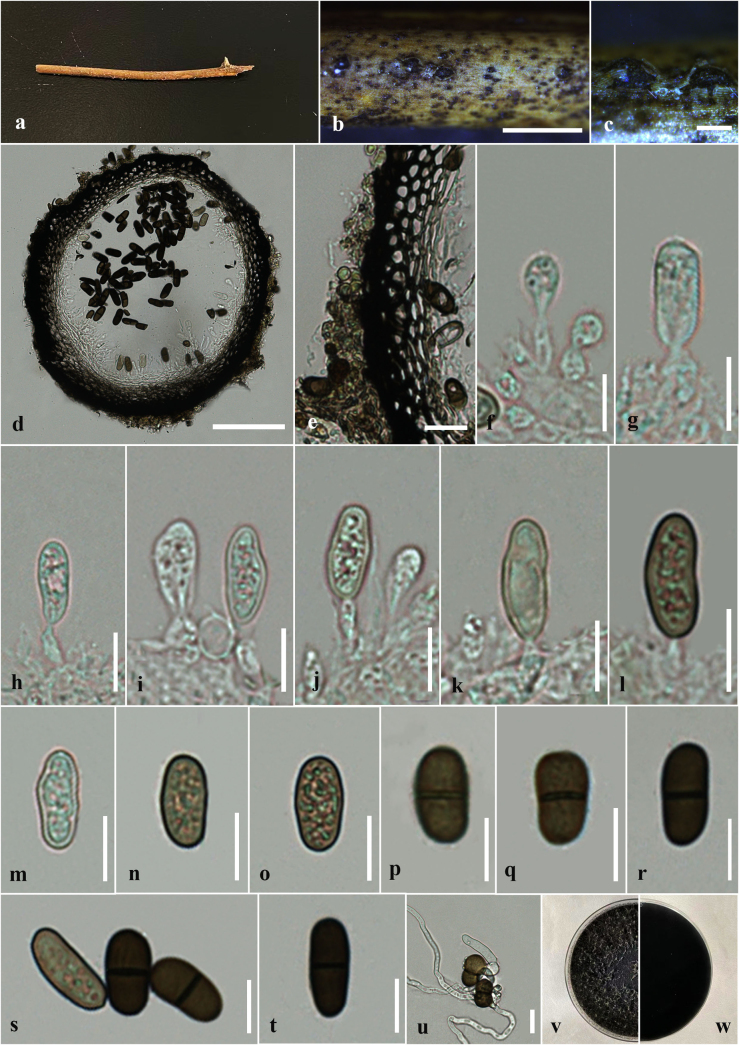
*Dothiorella fusiformis* (HKAS 151752, asexual morph) on dead twig of *Thalictrum* sp. **a**. Natural host substrate; **b, c**. Conidiomata on the host surface; **d**. A vertical section through conidioma; **e**. Peridium of conidioma; **f–l**. Conidia attached to conidiogenous cells; **m–t**. Conidia; **u**. Germinated conidia; **v, w**. Colony on PDA (**v**. Upper; **w**. Lower views). Scale bars: 1 mm (**b**); 200 μm (**c**); 100 μm (**d**); 20 μm (**e**); 10 μm (**f–u**).

Ascospores germinating on PDA within 24 h. Germ tubes produced at one side of ascospore. Colonies on PDA reaching 3–3.5 cm diam. after 4 days at 25 °C, colonies circular in shape, medium dense, flat or effuse, slightly raised, fluffy to fairly fluffy, aerial, white in middle and black in outer layer in both upper and lower sides (Fig. 3). Conidia germinating on PDA within 24 h. Germ tubes produced at one side of Conidia. Colonies on PDA reaching 2–3.5 cm diam. after 5 days at 25 °C, colonies circular in shape, medium dense, flat or effuse, slightly raised, fluffy to fairly fluffy, aerial, black in both upper and lower sides (Fig. 4).

##### Material examined.

China • Yunnan Province, Pu’er City, Xiaoheijiang Forest Park, lives on dead twigs of *Thalictrum* sp. (Ranunculaceae), 25 September 2025, Achala Rathnayaka, AS80 (HKAS 151748, **holotype**), ex-type living culture GBWSCCF120274; *ibid*., AS82 (HKAS 151752), living culture GBWSCCF120275.

##### Notes.

In the multi-gene phylogeny of combined LSU, ITS, *tef*1-α and *β-tubulin* analyses, our novel strains (GBWSCCF120274/HKAS 151748/GBWSCCF120275/HKAS 151752) formed a separate clade sister to *D. alpina* (CGMCC 3.18001) with 94% ML bootstrap and 0.98 PP support (Fig. 1, Group A). When comparing the basepair (bp) differences between our new isolate (HKAS 151748) and *D. alpina* (CGMCC 3.18001), ITS shows around 2.1% (10/478) differences (without gaps). Also, bp comparison between HKAS 151748/GBWSCCF120274 and HKAS 151752/GBWSCCF120275 revealed that 0.2% (1/490) and 0.1% (1/852) differences in the ITS and LSU, respectively. Phylogenetically closely related *Dothiorella alpina*, has been recorded only from its asexual morph by Hyde et al. (2020) in *Ipomoea* sp., Yunnan Province, China. Morphologically, the asexual morph of our new collection (HKAS 151752) differs from *D. alpina* (KUN-HKAS 102212) by the size of conidiomata (295–325 μm high × 310–375 μm diam. vs. 135–245 µm high × 150–235 μm diam.) (Hyde et al. 2020). In considering the conidial morphology, *D. fusiformis* has oblong to ellipsoidal, straight or slightly curved conidia that slightly constricted at the septum, guttulate and with l/w ratio of 2.5 (this study). However, in *D. alpina*, the conidia are oblong, occasionally irregular in shape, not constricted at the septa, and with l/w ratio of 2.19 (Hyde et al. 2020). Thus, based on distinct morphology and phylogenetic evidence, we established our new collection, *D. fusiformis* as a new species in *Dothiorella*. Also, in this study, we introduced both sexual (GBWSCCF120274/ HKAS 151748) and asexual (GBWSCCF120275/ HKAS 151752) morphs of *D. fusiformis*.

#### Dothiorella swieteniae

Taxon classificationFungiBotryosphaerialesBotryosphaeriaceae

Rathnayaka & F. Q. Yu sp. nov.

E7A94958-8685-5CDD-961C-5D84E96DFC1F

Index Fungorum: IF904782

Fig. 5

##### Etymology.

The epithet refers to the host genus “Swietenia”, from which the fungus was collected.

**Figure 5.**
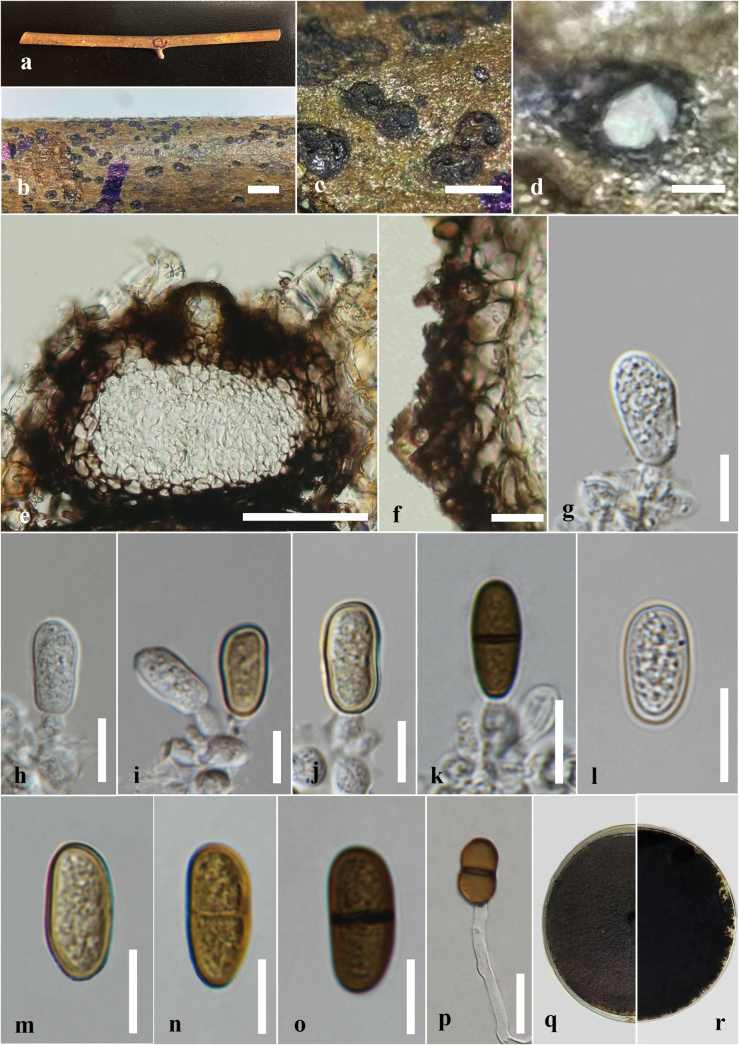
*Dothiorella swieteniae* (HKAS 151749, holotype) on dead branches of *Swietenia macrophylla*. **a**. Natural host substrate; **b, c**. Conidiomata on the host surface; **d, e**. A vertical section through conidioma; **f**. Peridium of conidioma; **g–k**. Conidia attached to conidiogenous cells; **l–o**. Conidia; **p**. A germinated conidium; **q, r**. Colony on PDA (**q**. Upper; **r**. Lower views). Scale bars: 1 mm (**b**); 500 μm (**c**); 100 μm (**d**); 50 μm (**e**); 10 μm (**f–p**).

##### Holotype.

HKAS 151749.

##### Description.

***Saprobic*** on dead branches of *Swietenia macrophylla* King. ***Sexual morph***: Undetermined. ***Asexual morph***: Coelomycetous. ***Conidiomata*** 110–125 μm high × 150–175 μm diam. (x̄ = 118 × 163 μm, n = 10) pycnidial, solitary, globose to sub globose, formed in uniloculate stromata, immersed, appear as black dots on host surface, ostiolate. ***Ostiole*** 30–40 μm diam., central, papillate. ***Conidiomatal wall*** 18–37 μm diam., 3–5 layers, consists of outer layers of thick-walled, dark brown to brown cells of *textura angularis*, inner layers consist of thin-walled, pale brown to hyaline cells of *textura angularis*. ***Conidiophores*** usually reduced to conidiogenous cells. ***Conidiogenous cells*** 4–9 μm high × 3–7 μm diam. (x̄ = 7 × 5 μm, n = 20), lining the pycnidial cavity, holoblastic, hyaline, cylindrical, discrete, determinate, smooth-walled. ***Conidia*** 14–28 μm high × 7–13 μm diam. (x̄ = 21 × 10 μm, n = 20, l/w = 2.1) ellipsoid, straight or slightly curved, rounded at both ends, initially hyaline and aseptate, becoming dark brown and 1-septate often while attached to conidiogenous cell, slightly constricted at the septum, guttulate.

##### Culture characteristics.

Conidia germinating on PDA within 24 h. Germ tubes produced at one side of Conidia. Colonies on PDA reaching 2–3.5 cm diam. after 6 days at 25 °C, colonies circular in shape, medium dense, flat or effuse, slightly raised, fluffy to fairly fluffy, aerial, black in both upper and lower sides.

##### Material examined.

China • Yunnan Province, Sun River National Forest Park, Pu’er City, lives on dead branches of *Swietenia macrophylla* (Meliaceae), 07 July 2025, Achala Rathnayaka, AS12 (HKAS 151749, **holotype**), ex-type living culture GBWSCCF120263.

##### Notes.

Morphologically, our new collection, *Dothiorella swieteniae* (HKAS 151749 and GBWSCCF120263) fits with the generic concept of *Dothiorella* in having 1-septate, brown conidia attached to the conidiogenous cells (Phillips et al. 2005; Dissanayake et al. 2016). Based on multigene phylogeny (LSU, ITS, *tef*1-α and *β-tubulin*) this novel taxon forms a distinct lineage basal to *Dothiorella* species in group A (Fig. 1), *i.e*., *Dothiorella acericola* (KUMCC 18-0137), *D. alpina* (CGMCC 3.18001), *D. citricola* (ICMP16828), *D. heterophyllae* (CMW46458), *D. hortiarborum* (CFCC 70756), *D. fusiformis* (GBWSCCF120274), *D. magnoliae* (CFCC 51563), *D. mangifericola* (CBS 121760), *D. plurivora* (IRAN1557C), *D. rosulata* (CBS 121760), *D. viticola* (WA10NO01), and *D. yunnana* (CGMCC 3.18000) with 85% ML and 0.90 PP statistical support values (Fig. 1). Detailed morphological comparison between species in group A and *D. swieteniae* is provided in Table 3. The l/w ratio (2.1) of conidia in *D. swieteniae* (HKAS 151749) is similar to *D. alpina*, *D. citricola* and *D. mangifericola* (Table 3). However, when considering the shape and verruculose surface of the conidia, *D. swieteniae* differs from *D. alpina*, *D. citricola* and *D. mangifericola* (Table 3). Thus, considering the morpho-molecular data analysis, we established *D. swieteniae* as a new species in *Dothiorella*.

**Table 3.** Synopsis of morphological characters of conidia among the *Dothiorella swieteniae* and species in group A.

Species	Conidia				Reference(s)
	Size (μm)	Average (μm)	Ratio (L/W)	Characters	
*Dothiorella acericola*	17–22(–23) × 7–10(–13)	20.8 × 9.2	2.2	Oblong to ellipsoidal, 1-septate, slightly constricted at the septum, smooth-walled	Phookamsak et al. (2019)
*D. alpina*	22–25(–28) × 10–12(–13)	24.4 × 11.1	2.1	Oblong with rounded ends, occasionally irregular in shape, 1-septate, smooth-walled	Hyde et al. (2020)
*D. citricola*	24–27 × 10–12	25.8 × 12.2	2.1	Oblong to subcylindrical, 1-septate, occasionally slightly constricted at septum, moderately thick walled, externally smooth, internally finely verruculose, ends truncate	Abdollahzadeh et al. (2014)
*D. heterophyllae*	18–26 × 8.5–11.5	22.6 × 9.8	2.3	Ellipsoidal, tapering towards a truncated base, 1-septate, septum median, verruculous	Jami et al. (2019)
*D. hortiarborum*	10.0–19.0 × 6.0–11.0	14.9 ± 2.6 × 8.1 ± 1.0	1.8	Ovoid with a broadly rounded apex, truncate base, uneven surface, thick-walled, 1-septate, constricted at the septum	Wu et al. (2024)
*D. fusiformis*	10–18 × 5–7	15 × 6	2.5	Oblong to ellipsoidal, straight or slightly curved, 1-septate slightly constricted at the septum, guttulate	This study
*D. magnoliae*	20.6–22.5 × 10.9–12.0	21.56 × 11.45	1.9	Internally finely verruculose, 1-septate, always deeply constricted at septum, externally smooth	You et al. (2017)
*D. mangifericola*	17–22 × 8–10	19 × 9	2.1	Subcylindrical to ellipsoid or ovoid, 1-septate, occasionally slightly constricted at septum, internally finely verruculose	Abdollahzadeh et al. (2014)
*D. plurivora*	(17–)18.7–20.6(–21) × (8–)8.5–9.7(–11)	19.7 × 9	2.2	Ellipsoid to ovoid, straight, 1-septate, slightly constricted at the septum, rounded at both ends	Hyde et al. (2024)
*D. rosulata*	(19–)21–24.2(–26.7) × (8–)10–10.5(–13)	22.6 × 10.4	2.2	Ovoid to subcylindrical or ellipsoidal, 1-septate, occasionally constricted at the septum, moderately thick-walled, smooth, mostly guttulate	Slippers et al. (2014)
*D. swieteniae*	14–28 × 7–13	21 × 10	2.1	Ellipsoid, straight or slightly curved, 1-septate, slightly constricted at the septum, guttulate	This study
*D. viticola*	(17.5–)19.5–23(–28.5) × (9–)10.5–12.5(–14)	21.3 × 11.4	1.9	Subcylindrical to ellipsoid or ovoid, 1-septate, occasionally slightly constricted at septum, moderately thick-walled, externally smooth, internally finely verruculose, ends rounded, often with a truncate base	Zhang et al. (2021)
*D. yunnana*	19.6–21 × 8.6–9.2	20.3 × 8.9	2.3	Ellipsoid, 1-septate, externally smooth, occasionally slightly constricted at the septum with rounded apex and truncate base	Zhang et al. (2016)

#### Dothiorella camelliae

Taxon classificationFungiBotryosphaerialesBotryosphaeriaceae

W.Li Li & Jian K. Liu (2023)

18850920-6357-5170-838E-875E16C0B893

Index Fungorum: IF847167

Fig. 6

##### Description.

***Saprobic*** on dead branches of *Macaranga peltata*. ***Sexual morph*: *Ascomata*** 125–150 × 100–225 μm (x̄ = 150 × 140 μm, n = 10), immersed to erumpent through host tissue, globose, solitary and scattered, black, multilocular or unilocular. ***Peridium*** 15–46 μm wide, composed of two layers, outer layer consists of thick-walled, brown to dark-brown cells of *textura angularis*, inner layer consists of thin-walled, pale brown to hyaline cells of *textura angularis*. ***Hamathecium*** comprising numerous 3–4 μm wide, hyaline, frequently aseptate pseudoparaphyses. ***Asci*** 72–90 × 20–25 μm (x̄ = 80 × 23 μm, n = 30), 8-spored, bitunicate, fissitunicate, cylindrical-clavate to clavate, short-pedicellate, apically rounded, with ocular chamber. ***Ascospores*** 18–25 × 9–12 μm (x̄ = 22 × 11 μm, n = 30, l/w ratio = 2), overlapping 2–3-seriate, oblong, ovate to sub-clavate, straight or slightly curved, initially hyaline and aseptate and becoming dark brown and 1-septate at maturity, slightly constricted at the septum, rough-walled. ***Asexual morph***: Undetermined.

**Figure 6.**
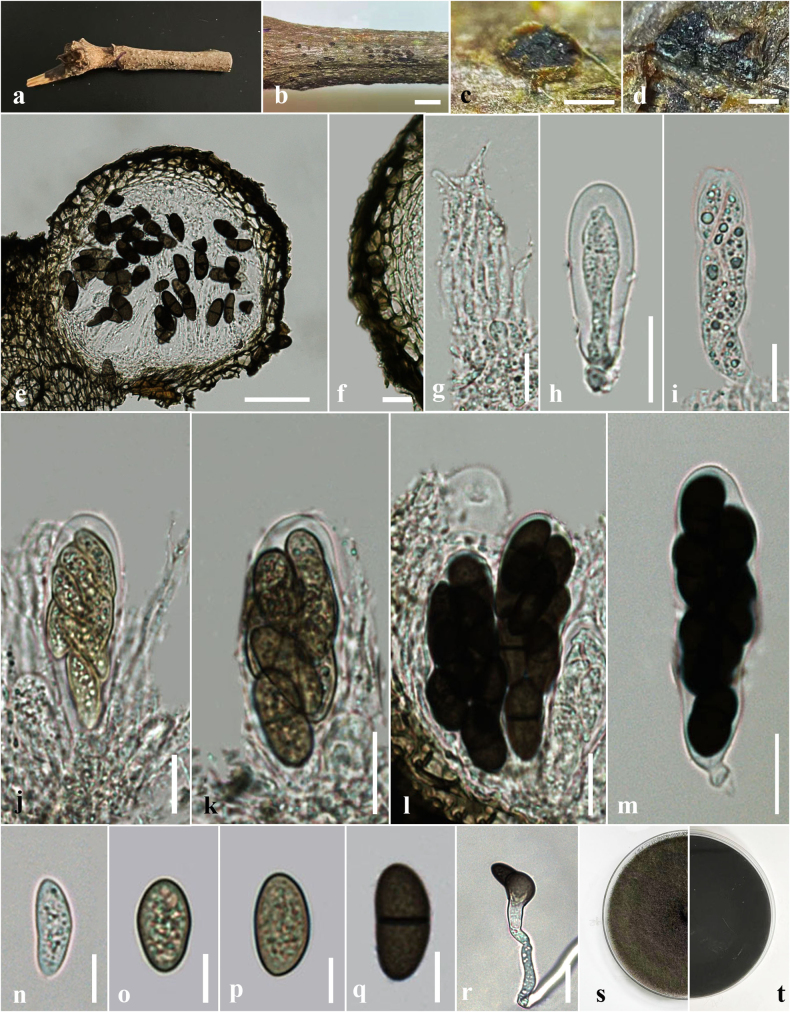
*Dothiorella camelliae* (HKAS 151751, a new host record) on dead branch of *Macaranga peltata*. **a**. Natural host substrate; **b, c**. Appearance of ascostromata on host; **d**. A section through ascostromata; **e**. A cross section of an ascoma; **f**. Peridium; **g**. Pseudoparaphyses; **h–m**. Asci; **n–q**. Ascospores; **r**. A germinated ascospore; **s, t**. Colony on PDA (**s**. Upper; **t**. Lower views). Scale bars: 2 mm (**b**); 500 μm (**c**); 200 μm (**d**); 50 μm (**e**); 20 μm (**h–m, r**); 10 μm (**f, g, n–q**).

##### Culture characteristics.

Ascospores germinating on PDA within 24 h. Germ tubes produced at one side of ascospore. Colonies on PDA reaching 2–3.5 cm diam. after 6 days at 25 °C, colonies circular in shape, medium dense, flat or effuse, slightly raised, fluffy to fairly fluffy, aerial, black in both upper and lower sides.

##### Material examined.

China • Yunnan Province, Pu’er City, lives on dead branches of *Macaranga peltata* Roxb. Mueller (Euphorbiaceae), 06 July 2025, Achala Rathnayaka, AS27 (HKAS 151751), living culture GBWSCCF110208.

##### Notes.

Based on multigene phylogenetic analyses (LSU, ITS, *tef*1-α and *β-tubulin*), our new strain (GBWSCCF110208) clustered with the ex-type strain of *D. camelliae* (CGMCC 3. 24158) with 100% ML and 1.00 PP statistical support (Fig. 1). Morphologically, our collection is similar to the holotype of *D. camelliae* (HKAS 125892), which was collected from decaying branches of *Camellia oleifera* in Sichuan Province, China (Li et al. 2023). Both strains have solitary or gregarious, globose, black, multilocular or unilocular ascomata; hyaline, aseptate pseudoparaphyses; clavate asci; and oblong, ovate to sub-clavate, dark brown ascospores (Li et al. 2023). Previously, *D. camelliae* was recorded on dead branches of *Camellia oleifera* and *Paeonia suffruticosa* in Sichuan and Shandong Provinces, China, respectively (Li et al. 2023). We determined our collection as a new host record of *D. camelliae* from *Macaranga peltata* in Yunnan Province, China.

#### Barriopsis archontophoenicis

Taxon classificationFungiBotryosphaerialesBotryosphaeriaceae

S. Konta, Boonmee & K.D. Hyde (2016)

8A3394D9-CC74-58DB-988B-82D347099668

Index Fungorum: IF552104

Fig. 7

##### Description.

***Saprobic*** on dead twig of *Clarisia racemosa*. ***Sexual morph*: *Ascomata*** 220–280 μm, high × 220–245 μm diam (x̄ = 260 × 225 μm, n = 10), solitary to aggregated, immersed, erumpent at maturity, uniloculate, subglobose, black, papillate. ***Peridium*** 25–60 μm wide, 3–5 layers, composed of outer layers of thick-walled, dark brown cells of *textura angularis*, inner layers composed of thin-walled, hyaline cells of *textura angularis*. ***Hamathecium*** comprising numerous 3–5 µm wide, hypha-like, septate, cellular pseudoparaphyses. ***Asci*** 75–140 × 20–34 μm (x̄ = 115 × 26 μm, n = 15), 8-spored, bitunicate, fissitunicate, cylindrical-clavate, short-pedicellate, apically rounded, with well development ocular chamber. ***Ascospores*** 23–30 × 8–11 μm (x̄ = 24 × 9.8 μm, n = 30, l/w = 2.4), overlapping 2–3-seriate, ellipsoid to ovoid, broad at the middle, aseptate, rough-walled, initially hyaline, with a large central guttule, becoming dark brown at maturity. ***Asexual morph***: Undetermined.

**Figure 7.**
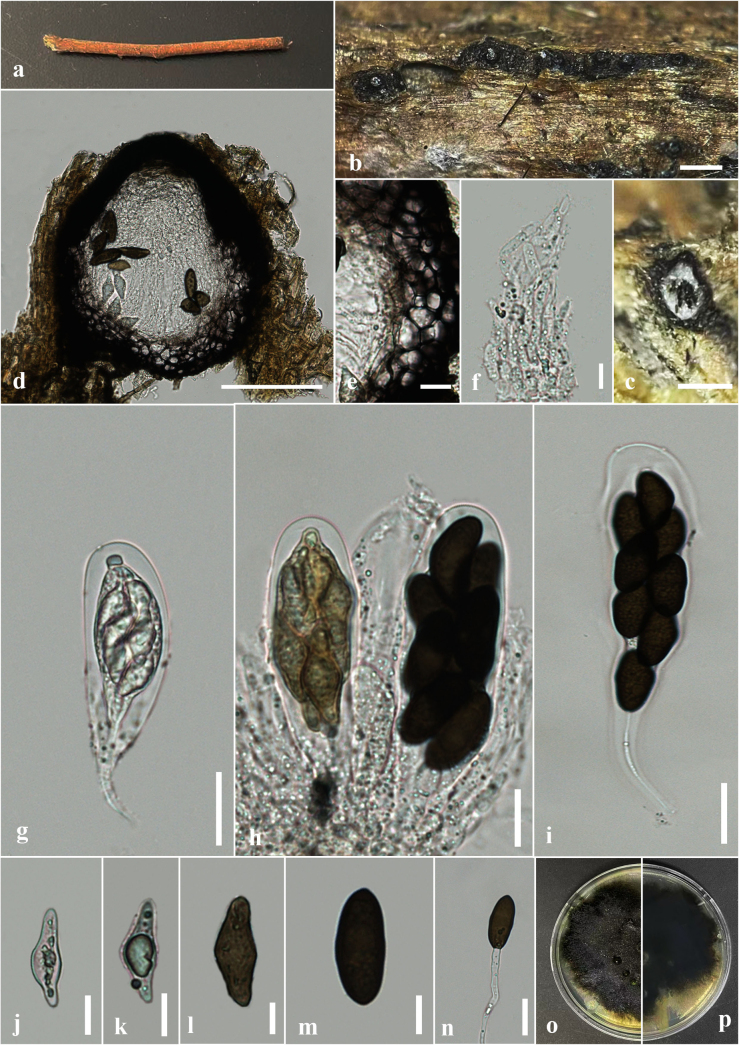
*Barriopsis archontophoenicis* (HKAS 151750, a new host record) on dead branch of *clarisia racemosa*. **a**. Natural host substrate; **b**. Appearance of ascostroma on the host substrate; **c, d**. A section through an ascoma; **e**. Peridium; **f**. Pseudoparaphyses; **g–i**. Asci; **j–m**. Ascospores; **n**. A germinated ascospore; **o, p**. Colony on PDA (**o**. Upper; **p**. Lower views). Scale bars: 200 μm (**b, c**); 100 μm (**d**); 20 μm (**g–i, n**); 10 μm (**e**, **f, j–m**).

##### Culture characters.

Ascospores germinating on PDA within 6 hours and germ tube produced from one end of the ascospore. Colonies on PDA reaching 6–8 cm diam. after 10 days at 25 °C, colonies irregular in shape, flat or effuse, slightly raised, pale yellow at the edge, black in both upper and lower sides.

##### Material examined.

China • Yunnan Province, Sun River National Forest Park, Pu’er City, on a dead twig of *Clarisia racemosa* (Moraceae), 07 July 2025, Achala Rathnayaka, AS14 (HKAS 151750), living culture GBWSCCF120264.

##### Notes.

The specimen examined in this study has similar morphology to the holotype of *Barriopsis archontophoenicis*, collected from *Archontophoenix alexandrae* in Thailand (Konta et al. 2016). Both isolates have solitary to aggregated, immersed ascomata, cylindrical-clavate, short-pedicellate, apically rounded asci, with a well-developed ocular chamber. Ascospores are ellipsoid to ovoid, broader at the middle, aseptate, and dark brown (Konta et al. 2016). Ascospore sizes are similar in both holotype and our collection (x̄ = 22 × 9 vs. x̄ = 24 × 9.8 μm, l/w = 2.4) (Konta et al. 2016). Based on the phylogenetic analyses, our strains of *B. archontophoenicis* (GBWSCCF120264 and HKAS 151750) clustered with the ex-type and other strain of *B. archontophoenicis* (MFLUCC 14-1164 and MFLU 20-0523) with 87% ML and 0.97 PP statistical support (Fig. 2). Previously, *B. archontophoenicis* have been recorded in *A. alexandrae* in Thailand (Konta et al. 2016) and *Camellia sinensis* in Taiwan province, China (Rathnayaka et al. 2021). Based on morpho-molecular analyses, we reported our collection as a new host record of *B. archontophoenicis* on *Clarisia racemosa* in China.

## Discussion

Saprobic fungi are ecologically important due to their critical role in decomposition and nutrient cycling. Ascomycota is considered one of the most representative fungal groups involved in saprobic behavior (Benny et al. 2001; Li et al. 2022). Investigating saprobic fungi is essential, as it helps in understanding their ecological roles and also discovering their hidden diversity (Du et al. 2025). Tropical forests have rich Ascomycota diversity, and Yunnan Province is dominated by tropical lower montane broad-leaved evergreen forests (Tedersoo et al. 2014; Zhu et al. 2021; Li et al. 2022). Therefore, Yunnan Province contains a rich diversity of Ascomycota. Feng and Yang (2018) mentioned that only around 6,000 of the estimated 104,000 fungal species have been described in Yunnan Province, and about 95% of the hidden fungal diversity still remain to be explored. Thus, studying saprobic Ascomycota in Yunnan Province is important.

Saprobic fungal species were collected from terrestrial habitats in Yunnan Province, China, from July – September 2025 from different forest areas, under tropical climatic conditions. Based on morphological characterization and molecular phylogeny, we introduced two new species and one new host record in *Dothiorella*; namely, *D. fusiformis*, *D. swieteniae* and *D. camelliae*, respectively. We provide complete morphological descriptions for both sexual and asexual morphs for *D. fusiformis*. Additionally, *Barriopsis archontophoenicis* is described as a new host record from *Clarisia racemosa* in Yunnan Province, China.

The genus *Dothiorella* accommodates fungal species characterized by conidia that develop pigmentation while attached to their conidiogenous cells (Phillips et al. 2005). Concurrently, *Spencermartinsia* was introduced to include *Dothiorella*-like taxa that have apiculate ascospores (Phillips et al. 2008). However, apiculate ascospores were considered unreliable for generic delineation (Yang et al. 2017). Therefore, with the support of multi-gene phylogenetic analyses, *Spencermartinsia* was synonymized with *Dothiorella* (Dissanayake et al. 2016; Zhang et al. 2016; Yang et al. 2017). Later, Zhou et al. (2025) showed that *Spencermartinsia* is a synonym of *Dothiorella* based on their comprehensive phylogenetic analyses.

In the recent decade, several species of *Dothiorella* have been described in China, including *D. acericola* on dead hanging twigs of *Acer palmatum* (Phookamsak et al. 2019), *D. baihuashanensis* on dead branches of *Juniperus chinensis* (Lin et al. 2023), *D. camelliae* on decaying branches of *Camellia oleifera* (Li et al. 2023), *D. hortiarborum* on dead branches of *Fraxinus chinensis* (Wu et al. 2024), *D. magnoliae* on twigs and branches of *Magnolia grandiflora* (You et al. 2017), *D. pugeense* on dead branches of *Juglans regia* saprobes (Wang et al. 2025), and *D. zanthoxyli* on decaying branches of *Zanthoxylum bungeanum* (Li et al. 2023). Most of these taxa were isolated from dead or decaying woody materials and are recorded as saprobes, except *D. magnoliae* (recorded as pathogens).

Several *Dothiorella* species have been reported as plant pathogens in China. *Dothiorella alpina*, *D. citrimurcotticola*, and *D. plurivora* were isolated from citrus branch diseases (Xiao et al. 2021). *Dothiorella acericola* has been associated with branch cankers of *Ziziphus jujuba* in Beijing, China (Pan et al. 2021), and *D. sarmentorum* was reported to cause leaf spot on *Lavandula angustifolia* in China (Li et al. 2024). In addition, *D. vidmadera*, *D. yunnana*, and *D. zanthoxyli* were isolated from cankered branches of *Chaenomeles cathayensis*, *Rosa chinensis*, and *Diospyros lotus*, respectively (Zhou et al. 2025). However, documentation of *Dothiorella* species as endophytes in China is lacking. Within *Barriopsis*, only *B. menglaense* has been introduced from China, occurring on woody twigs of *Castanopsis mekongensis* (Dong et al. 2025).

Introducing both the sexual and asexual morph of a new fungal species ensures comprehensive identification and accurate taxonomic classification. This study led to the expansion of the taxonomic framework and ecological relationships of Botryosphaeriaceae taxa in China by revealing a new species and two new host records. Therefore, these findings contribute to advancing our understanding of fungal species diversity in China. However, further investigations are needed to discover the hidden diversity of Botryosphaeriaceae species in China.

## Supplementary Material

XML Treatment for Dothiorella fusiformis

XML Treatment for Dothiorella swieteniae

XML Treatment for Dothiorella camelliae

XML Treatment for Barriopsis archontophoenicis

## Additional information

### Use of AI

No use of AI was reported.

### Author ORCIDs

Achala R. Rathnayaka https://orcid.org/0000-0001-8498-2715

Jianwei Liu https://orcid.org/0000-0001-5683-5152

Zhuyue Yan https://orcid.org/0009-0001-5954-6839

Shimei Yang https://orcid.org/0009-0008-3637-4182

Jing Yuan https://orcid.org/0009-0007-6849-2653

Xiaofei Shi https://orcid.org/0000-0002-4346-9867

Shanping Wan https://orcid.org/0000-0002-0794-3701

Dongqin Dai https://orcid.org/0000-0001-8935-8807

Indunil C. Senanayake https://orcid.org/0000-0002-7165-2394

Fuqiang Yu https://orcid.org/0000-0001-7931-080X

### Data availability

All of the data that support the findings of this study are available in the main text.

## References

[B1] Abdollahzadeh J, Mohammadi Goltapeh E, Javadi A, Shams-Bakhsh M, Zare R, Phillips AJ (2009) *Barriopsis iraniana* and *Phaeobotryon cupressi*: two new species of the Botryosphaeriaceae from trees in Iran. Persoonia 23: 1–8. 10.3767/003158509X467552 PMC2802722 20198156

[B2] Abdollahzadeh J, Javadi A, Zare R, Phillips AJL (2014) A phylogenetic study of *Dothiorella* and *Spencermartinsia* species associated with woody plants in Iran, New Zealand, Portugal and Spain. Persoonia 32: 1–12. 10.3767/003158514X678606 PMC4150070 25264380

[B3] Barr ME (1987) Prodromus to class Loculoascomycetes. Published by the author, Amherst, Massachusetts.

[B4] Batista E, Lopes A, Alves A (2021) What do we know about Botryosphaeriaceae? An overview of a worldwide cured dataset. Forests 12(3): 1–18. 10.3390/f12030313

[B5] Benny GL, Humber RA, Morton JB (2001) Zygomycota: Zygomycetes. Systematics and Evolution (Vol. 7a). Springer, Berlin/Heidelberg, 113–146. 10.1007/978-3-662-10376-0_6

[B6] Burgess TI, Tan YP, Garnas J, Edwards J, Scarlett KA, Shuttleworth LA, Daniel R, Dann EK, Parkinson LE, Dinh Q, Shivas RG (2019) Current status of the Botryosphaeriaceae in Australia. Australasian Plant Pathology 48(1): 35–44. 10.1007/s13313-018-0577-5

[B7] Capella-Gutiérrez S, Silla-Martínez JM, Gabaldón T (2009) trimAl: A tool for automated alignment trimming in large-scale phylogenetic analyses. Bioinformatics 25(15): 1972–1973. 10.1093/bioinformatics/btp348 PMC2712344 19505945

[B8] Carbone I, Kohn LM (1999) A method for designing primer sets for speciation studies in filamentous ascomycetes. Mycologia 91(3): 553–556. 10.1080/00275514.1999.12061051

[B9] Chernomor O, Von Haeseler A, Minh BQ (2016) Terrace aware data structure for phylogenomic inference from supermatrices. Systematic Biology 65(6): 997–1008. 10.1093/sysbio/syw037 PMC5066062 27121966

[B10] Chethana KWT, Li X, Zhang W, Hyde KD, Yan J (2016) Trail of decryption of molecular research on Botryosphaeriaceae in woody plants. Phytopathologia Mediterranea 55(2): 147–171. 10.14601/Phytopathol_Mediterr-16230

[B11] Damm U, Fourie PH, Crous PW (2007) *Aplosporella prunicola*, a novel species of anamorphic *Botryosphaeriaceae*. Fungal Diversity 27: 35–43.

[B12] Denman S, Crous PW, Taylor JE, Kang JC, Pascoe I, Wingfield MJ (2000) An overview of the taxonomic history of *Botryosphaeria* and a re-evaluation of its anamorphs based on morphology and ITS rDNA phylogeny. Studies in Mycology 45: 129–140.

[B13] Dissanayake AJ, Camporesi E, Hyde KD, Phillips AJL, Fu CY, Yan JY, Li XH (2016) *Dothiorella* species associated with woody hosts in Italy. Mycosphere 7(1): 51–63. 10.5943/mycosphere/7/1/6

[B14] Dong W, Hyde KD, Jeewon R, Karunarathna SC, Zhang H, Rossi W, Leonardi M, Kezo K, Kaliyaperumal M, Shu YX, Yang CL (2025) Fungal diversity notes 2017–2122: taxonomic and phylogenetic contributions to freshwater fungi and other fungal taxa. Fungal Diversity 134: 1–275. 10.1007/s13225-025-00560-3

[B15] Du TY, Karunarathna SC, Hyde KD, Nilthong S, Mapook A, Dai DQ, Rajeshkumar KC, Elgorban AM, Han LS, Wang HH, Tibpromma S (2025) New *Aquilariomyces* and *Mangifericomes* species (Pleosporales, Ascomycota) from *Aquilaria* spp. in China. MycoKeys 112: 103–125. 10.3897/mycokeys.112.139831 PMC11747782 39839667

[B16] Feng B, Yang Z (2018) Studies on diversity of higher fungi in Yunnan, southwestern China: A review. Plant Diversity 40(4): 165–171. 10.1016/j.pld.2018.07.001 PMC6137262 30740561

[B17] Garcia JF, Lawrence DP, Morales-Cruz A, Travadon R, Minio A, Hernandez-Martinez R, Rolshausen PE, Baumgartner K, Cantu D (2021) Phylogenomics of plant-associated *Botryosphaeriaceae* species. Frontiers in Microbiology 12: 1–18. 10.1101/2021.01.12.426103 PMC8012773 33815343

[B18] Glass NL, Donaldson GC (1995) Development of primer sets designed for use with the PCR to amplify conserved genes from filamentous ascomycetes. Applied and Environmental Microbiology 61(4): 1323–1330. 10.1128/aem.61.4.1323-1330.1995 PMC167388 7747954

[B19] Hall TA (1999) BioEdit: a user-friendly biological sequence alignment editor and analysis program for Windows 95/98/NT. Nucleic Acids Symposium Series 41: 95–98.

[B20] Hongsanan S, Hyde KD, Phookamsak R, Wanasinghe DN, McKenzie EHC, Sarma VV, Lücking R, Boonmee S, Bhat JD, Liu NG, Tennakoon DS, Pem D, Karunarathna A, Jiang SH, Jones GEB, Phillips AJL, Manawasinghe IS, Tibpromma S, Jayasiri SC, Sandamali D, Jayawardena RS, Wijayawardene NN, Ekanayaka AH, Jeewon R, Lu YZ, Phukhamsakda C, Dissanayake AJ, Zeng XY, Luo ZL, Tian Q, Thambugala KM, Dai D, Samarakoon MC, Chethana KWT, Ertz D, Doilom M, Liu JK, Pérez-Ortega S, Suija A, Senwanna C, Wijesinghe SN, Niranjan M, Zhang S-N, Ariyawansa HA, Jiang HB, Zhang J-F, Norphanphoun C, de Silva NI, Thiyagaraja V, Zhang H, Bezerra JDP, Miranda-González R, Aptroot A, Kashiwadani H, Harishchandra D, Sérusiaux E, Abeywickrama PD, Bao D-F, Devadatha B, Wu H-X, Moon KH, Gueidan C, Schumm F, Bundhun D, Mapook A, Monkai J, Bhunjun CS, Chomnunti P, Suetrong S, Chaiwan N, Dayarathne MC, Yang J, Rathnayaka AR, Xu J-C, Zheng JS, Liu G, Feng Y, Xie N (2020) Refined families of Dothideomycetes: orders and families incertae sedis in Dothideomycetes. Fungal Diversity 105(1): 17–318. 10.1007/s13225-020-00462-6

[B21] Hyde KD, de Silva NI, Jeewon R, Bhat DJ, Phookamsak R, Doilom M, Boonmee S, Jayawardena RS, Maharachchikumbura SSN, Senanayake IC, Manawasinghe IS, Liu NG, Abeywickrama PD, Chaiwan N, Karunarathna A, Pem D, Lin CG, Sysouphanthong P, Luo ZL, Wei DP, Wanasinghe DN, Norphanphoun C, Tennakoon DS, Samarakoon MC, Jayasiri SC, Jiang HB, Zeng XY, Li JF, Wijesinghe SN, Devadatha B, Goonasekara ID, Brahmanage RS, Yang EF, Aluthmuhandiram JVS, Dayarathne MC, Marasinghe DS, Li WJ, Dissanayake LS, Dong W, Huanraluek N, Lumyong S, Liu JK, Karunarathna SC, Jones EBG, Al-Sadi AM, Xu JC, Harishchandra D, Sarma VV, Bulgakov TS (2020) AJOM new records and collections of fungi: 1–100. Asian Journal of Mycology 3(1): 22–294. 10.5943/ajom/3/1/3

[B22] Hyde KD, Wijesinghe SN, Afshari N, Aumentado HD, Bhunjun CS, Boonmee S, Camporesi E, Chethana KWT, Doilom M, Dong W, Du TY, Farias ARG, Gao Y, Jayawardena RS, Karimi O, Karunarathna SC, Kularathnage ND, Lestari AS, Li CJY, Li YX, Liao CF, Liu XF, Lu L, Lu YZ, Luo ZL, Ma J, Mamarabadi M, Manawasinghe IS, Mapook A, Mi LX, Niranjan M, Senanayake IC, Shen HW, Su HL, Tibpromma S, Xu RJ, Yan JY, Yang YH, Yang YY, Yu FQ, Kang JC, Zhang JY (2024) Mycosphere Notes 469–520. Mycosphere 15(1): 1294–1454. 10.5943/mycosphere/15/1/11

[B23] Index Fungorum (2026) Index Fungorum. http://www.indexfungorum.org/names/Names.asp [accessed 10 January 2026]

[B24] Jami F, Marincowitz S, Slippers B, Crous PW, Le Roux JJ, Richardson DM, Wingfield MJ (2019) Botryosphaeriaceae associated with *Acacia heterophylla* (La Réunion) and *Acacia koa* (Hawaii). Fungal Biology 123(11): 783–790. 10.1016/j.funbio.2019.07.001 31627854

[B25] Jayawardena RS, Hyde KD, McKenzie EH, Jeewon R, Phillips AJ, Perera RH, de Silva NI, Maharachchikumburua SS, Samarakoon MC, Ekanayake AH, Tennakoon DS, Dissanayake AJ, Norphanphoun C, Lin C, Manawasinghe IS, Tian Q, Brahmanage R, Chomnunti P, Hongsanan S, Jayasiri SC, Halleen F, Bhunjun CS, Karunarathna A, Wang Y (2019) One stop shop III: taxonomic update with molecular phylogeny for important phytopathogenic genera: 51–75 (2019). Fungal Diversity 98: 1–84. 10.1007/s13225-019-00433-6

[B26] Katoh K, Rozewicki J, Yamada KD (2019) MAFFT online service: multiple sequence alignment, interactive sequence choice and visualization. Briefings in Bioinformatics 20: 1160–1166. 10.1093/bib/bbx108 PMC6781576 28968734

[B27] Konta S, Phillips AJL, Bahkali AH, Jones EBG, Eungwanichayapant DP, Hyde KD, Boonmee S (2016) Botryosphaeriaceae from palms in Thailand – *Barriopsis archontophoenicis* sp. nov, from *Archontophoenix alexandrae*. Mycosphere 7: 921–932. 10.5943/mycosphere/si/1b/1

[B28] Lazzizera C, Frisullo S, Alves A, Lopes J, Phillips AJL (2008) Phylogeny and morphology of *Diplodia* species on olives in southern Italy and description of *Diplodia olivarum*. Fungal Diversity 31: 63–71.

[B29] Li M, Liu C, Shi W, Wang A, Ma R, Su X (2024) Identification and pathogenicity of *Dothiorella sarmentorum* causing lavender leaf blight disease in Xinjiang, China. Diversity 16(3): e148. 10.3390/d16030148

[B30] Li WL, Liang RR, Dissanayake AJ, Liu JK (2023) Botryosphaerialean fungi associated with woody oil plants cultivated in Sichuan Province China. MycoKeys 97: 71–116. 10.3897/mycokeys.97.103118 PMC10230375 37265995

[B31] Li W, Liu J, Bhat DJ, Camporesi E, Xu J, Hyde KD (2014) Introducing the novel species, *Dothiorella symphoricarposicola*, from snowberry in Italy. Cryptogamie, Mycologie 35(3): 257–270. 10.7872/crym.v35.iss3.2014.257

[B32] Li X, Qu Z, Zhang Y, Ge Y, Sun H (2022) Soil fungal community and potential function in different forest ecosystems. Diversity 14(7): 1–12. 10.3390/d14070520

[B33] Lin L, Bai Y, Pan M, Tian C, Fan X (2023) Morphology and molecular analyses reveal three new species of Botryosphaeriales isolated from diseased plant branches in China. MycoKeys 97: 1–19. 10.3897/mycokeys.97.102653 PMC10194845 37214759

[B34] Liu JK, Phookamsak R, Doilom M, Wikee S, Li YM, Ariyawansha HA, Boonmee S, Chomnunti P, Dai DQ, Bhat DJ, Romero AI, Zhuang WY, Monkai J, Jones EBG, Chukeatirote E, Ko TWK, Zhao YC, Wang Y, Hyde KD (2012) Towards a natural classification of Botryosphaeriales. Fungal Diversity 57: 149–210. 10.1007/s13225-012-0207-4

[B35] Marincowitz S, Groenewald JZ, Wingfield MJ, Crous PW (2008) Species of Botryosphaeriaceae occurring on Proteaceae. Persoonia 21: 111–118. 10.3767/003158508X372387 PMC2846136 20396581

[B36] Mohali S, Slippers B, Wingfield MJ (2007) Identification of Botryosphaeriaceae from *Euclyptus*, *Acacia* and *Pinus* in Venezuela. Fungal Diversity 25: 103–125.

[B37] Nguyen LT, Schmidt HA, Von Haeseler A, Minh BQ (2015) IQ-TREE: a fast and effective stochastic algorithm for estimating maximum-likelihood phylogenies. Molecular Biology and Evolution 32(1): 268–274. 10.1093/molbev/msu300 PMC4271533 25371430

[B38] Nylander JAA (2004) MrModeltest 2.0. Program distributed by the author. Evolutionary Biology Centre, Uppsala University: 2–4.

[B39] Pan M, Lin L, Tian C, Fan X (2021) Two fungal species associated with canker disease of Jujube tree in China. MycoAsia 3: 1–21. 10.59265/mycoasia.2021-03

[B40] Phillips AJL, Alves A (2009) Taxonomy, phylogeny, and epitypification of *Melanops tulasnei*, the type species of *Melanops*. Fungal Diversity 38: 155–166.

[B41] Phillips A, Alves A, Correia A, Luque J (2005) Two new species of *Botryosphaeria* with brown, 1- septate ascospores and *Dothiorella* anamorphs. Mycologia 97: 513–529. 10.1080/15572536.2006.11832826 16396358

[B42] Phillips AJL, Alves A, Abdollahzadeh J, Slippers B, Wingfield MJ, Groenewald JZ, Crous PW (2013) The Botryosphaeriaceae: genera and species known from culture. Studies in Mycology 76(1): 51–167. 10.3114/sim0021 PMC3825232 24302790

[B43] Phillips AJL, Alves A, Pennycook SR, Johnston PR, Ramaley A, Akulov A, Crous PW (2008) Resolving the phylogenetic and taxonomic status of dark-spored teleomorph genera in the Botryosphaeriaceae. Persoonia 21: 29–55. 10.3767/003158508X340742 PMC2846129 20396576

[B44] Phillips AJL, Hyde KD, Alves A, Liu JK (2019) Families in Botryosphaeriales: a phylogenetic, morphological and evolutionary perspective. Fungal Diversity 94: 1–22. 10.1007/s13225-018-0416-6

[B45] Phookamsak R, Hyde KD, Jeewon R, Bhat DJ, Jones EBG, Maharachchikumbura SSN, Raspé O, Karunarathna SC, Wanasinghe DN, Hongsanan S, Doilom M, Tennakoon DS, Machado AR, Firmino AL, Ghosh A, Karunarathna A, Mešić A, Dutta AK, Thongbai B, Devadatha B, Norphanphoun C, Senwanna C, Wei D, Pem D, Ackah FK, Wang GN, Jiang H-B, Madrid H, Lee HB, Goonasekara ID, Manawasinghe IS, Kušan I, Cano J, Gené J, Li J, Das K, Acharya K, Raj KNA, Latha KPD, Chethana KWT, He MQ, Dueñas M, Jadan M, Martín MP, Samarakoon MC, Dayarathne MC, Raza M, Park MS, Telleria MT, Chaiwan N, Matočec N, de Silva NI, Pereira OL, Singh PN, Manimohan P, Uniyal P, Shang Q-J, Bhatt RP, Perera RH, Alvarenga RLM, Nogal-Prata S, Singh SK, Vadthanarat S, Oh S-Y, Huang S-K, Rana S, Konta S, Paloi S, Jayasiri SC, Jeon SJ, Mehmood T, Gibertoni TB, Nguyen TTT, Singh U, Thiyagaraja V, Sarma VV, Dong W, Yu X-D, Lu YZ, Lim YW, Chen Y, Tkalčec Z, Zhang ZF, Luo Z-L, Daranagama DA, Thambugala KM, Tibpromma S, Camporesi E, Bulgakov TS, Dissanayake AJ, Senanayake IC, Dai DQ, Tang L-Z, Khan S, Zhang H, Promputtha I, Cai L, Chomnunti P, Zhao R-L, Lumyong S, Boonmee S, Wen T-C, Mortimer PE, Xu J (2019) Fungal diversity notes 929–1035: taxonomic and phylogenetic contributions on genera and species of fungi. Fungal Diversity 95: 1–273. 10.1007/s13225-019-00421-w

[B46] Rambaut A (2012) Fig.Tree. Tree Fig. Drawing Tool, v. 1.4.0. http://tree.bio.ed.ac.uk/software/figtree/ [Accessed November 1, 2025]

[B47] Rathnayaka AR, Chethana KWT, Phillips AJ, Jones EG (2022) Two new species of Botryosphaeriaceae (Botryosphaeriales) and new host/geographical records. Phytotaxa 564(1): 8–38. 10.11646/phytotaxa.564.1.2

[B48] Rathnayaka AR, Chethana KWT, Phillips AJ, Liu JK, Hyde KD (2021) First report of Botryosphaeriaceae species on *Camellia sinensis* from Taiwan with a global checklist of Botryosphaeriaceae species on this host. Chiang Mai Journal of Sciences 48(5): 1199–1223.

[B49] Rathnayaka AR, Tennakoon DS, Jones GE, Wanasinghe DN, Bhat DJ, Priyashantha AH, Stephenson SL, Tibpromma S, Karunarathna SC (2024) Significance of precise documentation of hosts and geospatial data of fungal collections, with an emphasis on plant-associated fungi. New Zealand Journal of Botany 63(2–3): 462–489. 10.1080/0028825X.2024.2381734

[B50] Rojas EI, Herre EA, Mejía LC, Arnold AE, Chaverri P, Samuels GJ (2008) *Endomelanconiopsis*, a new anamorph genus in the *Botryosphaeriaceae*. Mycologia 100(5): 760–775. 10.3852/07-207 18959162

[B51] Ronquist F, Teslenko M, Van Der Mark P, Ayres DL, Darling A, Höhna S, Larget B, Liu L, Suchard MA, Huelsenbeck JP (2012) Mrbayes 3.2: Efficient bayesian phylogenetic inference and model choice across a large model space. Systematic Biology 61: 539–542. 10.1093/sysbio/sys029 PMC3329765 22357727

[B52] Saccardo PA (1880) Conspectus genera fungorum Italiae inferiorum nempe ad Sphaeropsideas, Melanconieas et Hyphomyceteas pertinentium systemate sporologico dispositorum. Michelia 2: 1–38.

[B53] Schoch CL, Shoemaker RA, Seifert KA, Hambleton S, Spatafora JW, Crous PW (2006) A multigene phylogeny of the Dothideomycetes using four nuclear loci. Mycologia 98: 1041–1052. 10.1080/15572536.2006.11832632 17486979

[B54] Senanayake IC, Rathnayaka AR, Marasinghe DS, Calabon MS, Gentekaki E, Lee HB, Hurdeal VG, Pem D, Dissanayake LS, Wijesinghe SN, Bundhun D, Nguyen TT, Goonasekara ID, Abeywickrama PD, Bhunjun CS, Jayawardena RS, Wanasinghe DN, Jeewon R, Bhat DJ, Xiang MM (2020) Morphological approaches in studying fungi: collection, examination, isolation, sporulation and preservation. Mycosphere 11: 2678–2754. 10.5943/mycosphere/11/1/20

[B55] Slippers B, Wingfield MJ (2007) Botryosphaeriaceae as endophytes and latent pathogens of woody plants: diversity, ecology and impact. Fungal Biology Reviews 21(2–3): 90–106. 10.1016/j.fbr.2007.06.002

[B56] Slippers B, Boissin E, Phillips AJL, Groenewald JZ, Lombard L, Wingfield MJ, Postma A, Burgess T, Crous PW (2013) Phylogenetic lineages in the Botryosphaeriales: a systematic and evolutionary framework. Studies in Mycology 76: 31–49. 10.3114/sim0020 PMC3825231 24302789

[B57] Slippers B, Roux J, Wingfield MJ, Van der Walt FJJ, Jami F, Mehl JWM, Marais GJ (2014) Confronting the constraints of morphological taxonomy in the Botryosphaeriales. Persoonia-Molecular Phylogeny and Evolution of Fungi 33(1): 155–168. 10.3767/003158514X684780 PMC4312931 25737598

[B58] Tedersoo L, Bahram M, Põlme S, Kõljalg U, Yorou NS, Wijesundera R, Ruiz LV, Vasco-Palacios AM, Thu PQ, Suija A, Smith ME, Sharp C, Saluveer E, Saitta A, Rosas M, Riit T, Ratkowsky D, Pritsch K, Põldmaa K, Piepenbring M, Phosri C, Peterson M, Parts K, Pärte K, Otsing E, Nouhra E, Njouonkou AL, Nilsson RH, Morgado LN, Mayor J, May TW, Majuakim L, Lodge DJ, Lee SS, Larsson K-H, Kohout P, Hosaka K, Hiiesalu I, Henkel TW, Harend H, Guo L-D, Greslebin A, Grelet G, Geml J, Gates G, Dunstan W, Dunk C, Drenkhan R, Dearnaley J, Kesel AD, Dang T, Chen X, Buegger F, Brearley FQ, Bonito G, Anslan S, Abell S, Abarenkov K (2014) Global diversity and geography of soil fungi. Science 346(6213): e1256688. 10.1126/science.1256688 25430773

[B59] Theissen F, Sydow H (1918) Vorentwu¨rfe zu den Pseudosphaeriales. Annales Mycologici 16: 1–34.

[B60] Vilgalys R, Hester M (1990) Rapid genetic identification and mapping of enzymatically amplified ribosomal DNA from several *Cryptococcus* species. Journal of Bacteriology 172: 4238–4246. 10.1128/jb.172.8.4238-4246.1990 PMC213247 2376561

[B61] Wang FH, Xu XL, Zeng Q, Liu LJ, Liu F, Deng Y, Sun QR, Yan YQ, Li XY, Xiang SS, Shuai Q (2025) Microfungi associated with walnut trees in southwestern China. Mycosphere 16(1): 2002–2258. 10.5943/mycosphere/16/1/12

[B62] White TJ, Bruns T, Lee S, Taylor J (1990) Amplification and direct sequencing of fungal ribosomal RNA genes for phylogenetics. In: Innis MA, Gelfand DH, Sninsky JJ, White TJ (Eds) PCR protocols: a guide to methods and applications. Academic Press, Inc., New York, 315–322. 10.1016/B978-0-12-372180-8.50042-1

[B63] Wijayawardene NN, Hyde KD, Dai DQ, Sánchez-García M, Goto BT, Saxena RK, Erdoðdu M, Selçuk F, Rajeshkumar KC, Aptroot A, Błaszkowski J, Boonyuen N, da Silva GA, de Souza FA, Dong W, Ertz D, Haelewaters D, Jones EBG, Karunarathna SC, Kirk PM, Kukwa M, Kumla J, Leontyev DV, Lumbsch HT, Maharachchikumbura SSN, Marguno F, Martínez-Rodríguez P, Mešić A, Monteiro JS, Oehl F, Pawłowska J, Pem D, Pfliegler WP, Phillips AJL, Pošta A, He MQ, Li JX, Raza M, Sruthi OP, Suetrong S, Suwannarach N, Tedersoo L, Thiyagaraja V, Tibpromma S, Tkalčec Z, Tokarev YS, Wanasinghe DN, Wijesundara DSA, Wimalaseana SDMK, Madrid H, Zhang GQ, Gao Y, Sánchez-Castro I, Tang LZ, Stadler M, Yurkov A, Thines M (2022) Outline of Fungi and fungus-like taxa – 2021. Mycosphere 13: 53–453. 10.5943/mycosphere/13/1/2

[B64] Wu Y, Peng C, Yuan R, Zhang M, Hu Y, Tian C (2024) New species and records of Botryosphaeriales (Dothideomycetes) associated with tree dieback in Beijing, China. MycoKeys 106: 225–250. 10.3897/mycokeys.106.122890 PMC11224674 38974461

[B65] Xiao XE, Wang W, Crous PW, Wang HK, Jiao C, Huang F, Pu ZX, Zhu ZR, Li HY (2021) Species of Botryosphaeriaceae associated with citrus branch diseases in China. Persoonia-Molecular Phylogeny and Evolution of Fungi 47(1): 106–135. 10.3767/persoonia.2021.47.03 PMC10486630 37693792

[B66] Yang T, Groenewald JZ, Cheewangkoon R, Jami F, Abdollahzadeh J, Lombard L, Crous PW (2017) Families, genera, and species of Botryosphaeriales. Fungal Biology 121(4): 322–346. 10.1016/j.funbio.2016.11.001 28317538

[B67] You CJ, Liu X, Li LX, Tsui CKM, Tian CM (2017) *Dothiorella magnoliae*, a new species associated with dieback of *Magnolia grandiflora* from China. Mycosphere 8(2): 1031–1041. 10.5943/mycosphere/8/2/6

[B68] Zhang M, He W, Wu JR, Zhang Y (2016) Two new species of *Spencermartinsia* (Botryosphaeriaceae, Botryosphaeriales) from China. Mycosphere 7(7): 942–949. 10.5943/mycosphere/si/1b/4

[B69] Zhang W, Groenewald JZ, Lombard L, Schumacher RK, Phillips AJL, Crous PW (2021) Evaluating species in Botryosphaeriales. Persoonia-Molecular Phylogeny and Evolution of Fungi 46: 63–115. 10.3767/persoonia.2021.46.03 PMC9311389 35935886

[B70] Zhang X, Tibpromma S, Karunarathna SC, Du TY, Han LS, Elgorban AM, Kumla J, Senwanna C, Dai DQ, Suwannarach N, Wang HH (2025) Additions to the saprobic fungi (Ascomycota) associated with macadamia trees from the Greater Mekong Subregion. MycoKeys 113: 1–29. 10.3897/mycokeys.113.140031 PMC11786193 39897715

[B71] Zhou J, Li A, Jiang N (2025) Morphology and phylogeny reveal new species and records of *Diplodia*, *Dothiorella*, and *Phaeobotryon* associated with tree cankers in Xizang, China. Journal of Fungi 11(5): 1–19. 10.3390/jof11050331 PMC12112754 40422665

[B72] Zhou Y, Gong G, Cui Y, Zhang D, Chang X, Hu R, Liu N, Sun X (2015) Identification of Botryosphaeriaceae species causing kiwifruit rot in Sichuan Province, China. Plant Disease 99(5): 699–708. 10.1094/PDIS-07-14-0727-RE 30699681

[B73] Zhu H, Ashton P, Gu B, Zhou S, Tan Y (2021) Tropical deciduous forest in Yunnan, southwestern China: Implications for geological and climatic histories from a little-known forest formation. Plant Diversity 43(6): 444–451. 10.1016/j.pld.2021.01.001 PMC8720685 35024513

